# Effects of two contrasting dietary polysaccharides and tannic acid on the digestive and physicochemical properties of wheat starch

**DOI:** 10.1002/fsn3.2559

**Published:** 2021-08-31

**Authors:** Juncheng He, Lirong Zeng, Junan Gong, Yalun He, Xiong Liu, Ling Zhang, Na Xu, Qiong Wang

**Affiliations:** ^1^ College of Life Sciences and Health Wuhan University of Science and Technology Wuhan China

**Keywords:** *in vitro* digestibility, konjac glucomannan, tannic acid, wheat starch, κ‐carrageenan

## Abstract

In this study, konjac glucomannan, κ‐carrageenan, and tannic acid were selected to study the effects of different combinations on the in vitro digestibility and physicochemical properties of wheat starch. Results showed that the addition of konjac glucomannan, κ‐carrageenan, and tannic acid could decrease the digestion of starch and increase the content of resistant starch. Besides, the two polysaccharides weakened the extent of tannic acid on starch digestion. Moreover, although the two polysaccharides had different effects on the in vitro digestion of starch, they had no significant increase in the content of resistant starch. DSC and XRD results demonstrated that the polysaccharides and tannic acid showed synergistic effects on the rebuilding of starch microstructure. FTIR results further manifested that κ‐carrageenan and konjac glucomannan could significantly increase the strength of hydrogen bonds in starch. At the same time, the addition of tannic acid would weaken the molecular interaction between polysaccharides and starch. *SEM* and CLSM results showed that tannic acid added to the polysaccharide–starch mixture not only interacted with starch but also influenced the structure of polysaccharide gel.

## Graphical abstract

Two dietary polysaccharides inhibited the direct contact of tannic acid with starch. Polysaccharides and tannic acid synergistically inhibited the formation of ordered starch structure. Tannic acid weakened the molecular interaction between the two polysaccharides and wheat starch.

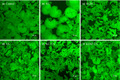

## 1 INTRODUCTION

Excessive intake of highly processed foods may led to a variety of metabolism‐related diseases (Lv et al., 2016; Roberts & Leibel, 1998; Svendsen & Tonstad, 2004). Compared with the treatment of traditional drugs, dietary polysaccharides and polyphenols can alleviate the metabolism‐related diseases with the advantages of little toxic and side effects (Darvesh et al., 2010; Qi et al., 2018; Solia et al., 2018; Soliman, 2019). As a result, increasing attention has been paid on the development of functional starchy foods by adding natural dietary ingredients (Brennan et al., 2004; Koh & Rowling, 2017).

Dietary polysaccharides and polyphenols have been widely used for improving the gelatinization, retrogradation, and microstructure of starch (Li et al., 2018; Raguzzoni et al., 2016; Sasaki & Kohyama, 2011). Many studies have shown that the interactions among dietary polysaccharides, polyphenols, and starch are mainly dependent on the molecular structure of polysaccharides (linear, branched, charged, or uncharged) and polyphenols (Gao et al., 2017; Zhu, 2015). However, it remains unclear how the interaction between polysaccharides and polyphenols with different molecular structures may affect the functional properties of starch.

Two contrasting dietary polysaccharides were selected in this study. Konjac glucomannan (KGM) is a nonionic polysaccharide, which formed by the main chain composed of D‐mannose and D‐glucose linked by β‐1,4 glycosidic bonds (Shah et al., 2015). It is reported that konjac glucomannan can affect the structure and physical properties of starch, thus improving the characteristics of starchy food (Lafarge & Cayot, 2018). In our previous work, konjac glucomannan addition is found to reduce the digestion of wheat starch through forming a barrier around the gelatinized starch molecule (Zhang et al., 2020). κ‐carrageenan (KC) is an anionic, linear polysaccharides extracted from red seaweed (Necas & Bartosikova, 2013). Studies have shown that the strength of interactions between κ‐carrageenan and starch in water depends on the types and quantities of negative charge of the polysaccharide (Lascombes et al., 2017).

Tannic acid is an important natural polyphenolic active substance widely present in plants. Due to abundant phenolic hydroxyl groups, tannic acid can interact with amino acid residues in the active site of digestive enzymes, thereby inhibiting its activity (Funke & Melzig, 2005; Li et al., 2019; Zhao et al., 2013). Also, the addition of tannic acid to wheat starch could spontaneously form complex, thus affecting the rheological properties and microstructure of starch (Wei et al., 2019). Our previous research has found that the gelatinization processing does not give negative effect but even promote the nutritional value of tannic acid in wheat starch such as phenolic content and antioxidant activity (Zeng et al., 2020). Therefore, the main objectives in the present work were to determine how the in vitro digestion, gelatinization, retrogradation, and microstructure of wheat starch were affected by different combinations of konjac glucomannan, κ‐carrageenan, and tannic acid.

## 2 MATERIALS AND METHODS

### 2.1 Materials

Konjac glucomannan were purchased from Wuhan Yangyoudao health industry co., LTD. (Hubei, China). κ*‐*Carrageenan was purchased from Shanghai Yuanye bio‐technology Co., Ltd. Wheat starch, tannic acid, 3, 5‐dinitrosalicylic acid (DNS), fluorescein isothiocyanate (FITC), pepsin (enzymatic activity 456 U/mg) derived from pig gastric mucosa, alpha‐amylase (enzymatic activity 1,000 ~ 3,000 U/mg protein) derived from human saliva, and amyloglucosidase (enzymatic activity 30 ~ 60 U/mg protein) derived from aspergillus niger were all purchased from Sigma‐Aldrich.

### 2.2 Sample processing

In the experimental groups, the addition ratio of konjac glucomannan (KGM) and κ‐carrageenan (KC) was 3%, and the addition ratio of tannic acid (TA) was 0.2%. The concentrations of KGM, KC, and TA were chosen according to the preliminary experiments and the reference (Zeng et al., 2020; Zhang et al., 2020). The mixing percentages of added substances were based on the mass ratio of wheat starch (*w/w*). Tannic acid was prepared into a solution of 0.01 g/ml and stored at 4℃.

Wheat starch (2 g) was accurately weighed, added with 30 ml distilled water, and prepared in a centrifuge tube. After mixed well with the additions, the samples were shaken in the water bath at 95℃ for 20 min. The gelatinized mixtures were prefrozen for 24 h in a −20℃ freezer and dried for 48 h in a freeze dryer (science‐12N; Ningbo Xingzhi Biotechnology Co., Ltd.) at −50℃ (Xie et al., 2018). After freeze‐drying, the samples were crushed and screened with 100 mesh. The powder samples were stored in a vacuum drying centrifugal tube at 4℃.

### 2.3 Measurement of in vitro digestibility

In vitro digestion of wheat starch was conducted following the measurement described by Se˛czyk et al. (2017) with minor modifications. The brief steps were described below: Firstly, wheat starch (200 mg) was dispersed in 2 ml distilled water, mix well, and shaken in the water bath at 95℃ for 20 min. After the samples cooling to room temperature, 10 ml pepsin solution (0.01 M, KCl‐HCl buffer, PH = 1.2) was added and incubated at 37℃ for 1 h. Next, the volumes of the samples were replenished to 25 ml with sodium phosphate buffer (PH = 7), and each group was added with 5 ml alpha‐amylase buffer (2 U/ml) and incubated at 37℃ for 2 h. At the 10 min, 20 min, 30 min, 40 min, 50 min, 60 min, 90 min, and 120 min of the reaction, 1 ml of the reaction solution was, respectively, taken into a new centrifugal tube to inactivate the enzyme in a metal bath at 100℃ for 5 min. After that, each tube was incubated for 1 h at 60℃ with 3 ml sodium acetate buffer (PH = 4.75) and 21 μl starch glycosidase solution (40 U/ml). At the end of incubation, 20 μl sample solution was taken, and the glucose content was determined by the DNS method (Saqib & Whitney, 2011). Finally, the amount of starch hydrolysis at each moment and the percentages of the rapidly digestible starch (RDS), the slowly digestible starch (SDS), and resistant starch (RS) were calculated by the following equations:

$$\text{RDS}\;\left(\%\right)=G20\times \frac{0.9}{\text{TS}}$$

$$\text{SDS}\left(\%\right)=\left(G120‐G20\right)\times \frac{0.9}{\text{TS}}$$

$$\text{RS}\left(\%\right)=\frac{\left[\text{TS}‐\left(\text{RDS}+\text{SDS}\right)\right]}{\text{TS}}$$

G20: Glucose content in hydrolysate for 20 min (mg).

G120: Glucose content in hydrolysate for 120 min (mg).

TS: Total starch content (mg).

### 2.4 Differential scanning calorimetry (DSC)

Differential scanning calorimetry (DSC‐7; Perkin Elmer, Norwalk, CT, USA) was used to assess the process of gelatinization. For each group, 100 mg wheat starch and 400 μl distilled water mixed well, and 10 μl of the mixture was taken out for testing. Before the thermal scanning, the sample was sealed in the gland and balanced at 4℃ for 12 h. Using an empty crucible as a reference, the prepared samples were heated from 20 to 120℃ at 10℃/min. The transition temperatures of the onset (T_o_), peak (T_p_), and endothermic enthalpy of gelatinization (ΔH) were recorded.

### 2.5 X‐ray diffraction (XRD)

X‐ray diffraction (XRD) patterns of the powder samples were measured with Cu‐Ka radiation using a Powder X‐ray diffractometer (Rigaku Mini Hex‐II, Japan). The region of scanning ranged from 10º to 70º, and the speed of goniometer was 4º/min operated at 40 mA and 40 kV (Sukhija et al., 2016). According to the X‐ray diffraction pattern, software Jade6 was used to calculate the relative crystallinity of each group (Jing et al., 2019; Xie et al., 2018).

### 2.6 Fourier transform infrared spectroscopy (FTIR)

The FTIR spectra were run using a TENSORⅡ spectrometer (Bruker Corporation). Two mg powder samples were dispersed in 200 mg KBr, which then was extracted by air for 1 min using a tablet pressing machine mold. The spectra were recorded in transmission mode from 4,000 to 400 cm^−1^ at room temperature.

### 2.7 Scanning electron microscopy (*SEM*)

Morphology of starch granules was evaluated by *SEM* (JSM 5,410 LV, Jeol Ltd.). After freeze‐drying, the samples were deposited directly on the aluminum pile with double‐sided carbon conductive tape and coated with a layer of gold. The images were obtained at 250 magnifications operated at 15 kV (Bridson et al., 2019; Jing et al., 2019).

### 2.8 Confocal light scanning microscopy (CLSM)

Two ml distilled water and 200 mg starches were mix well, and the samples were shaken in a water bath at 95℃ for 20 min. Five hundred μl of the gelatinized mixtures was stained by 20 μl FITC and incubated at 4℃ overnight. The fluorescent signal of stained starch granules was detected by oil lens of confocal laser scanning microscope (Olympus, Japan) under the field at 60 magnifications. The excitation wavelength was 488 nm, and the sizes of the images were 1,024/1,024 pixels. During image acquisition, each row was scanned four times and averaged to reduce noise (Chen et al., 2011).

### 2.9 Statistic analysis

Data were presented as means ± *SEM* of triplicate. Analysis of variance was used to contrast the significant difference by using one‐way analysis of variance (ANOVA). The significant differences (*p* < .05) were evaluated and displayed by different letters.

## 3 RESULTS

### 3.1 Effects of different combinations of konjac glucomannan, κ‐carrageenan, and tannic acid on the in vitro digestibility of wheat starch

**FIGURE 1:**
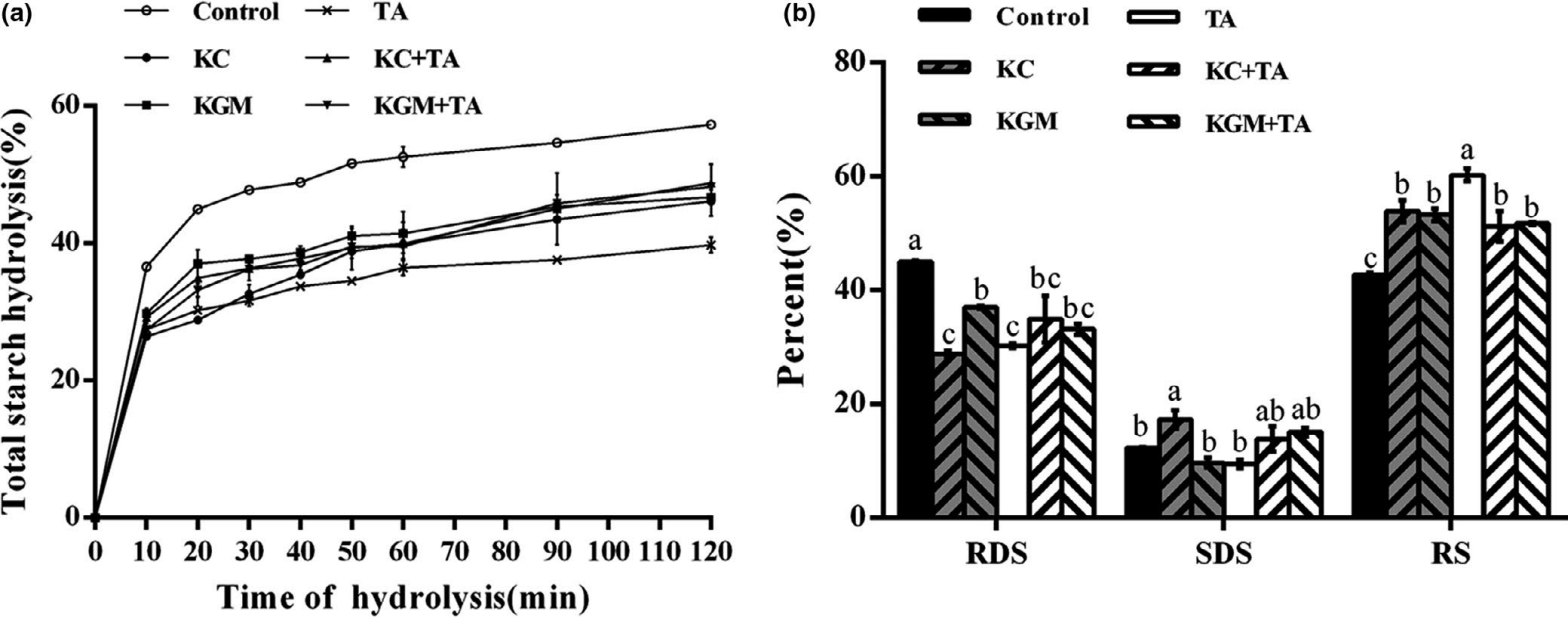
Effects of different combinations of konjac glucomannan, κ‐carrageenan, and tannic acid on starch in vitro digestion. (a) The hydrolysis percentage of wheat starch samples. (b) The content of RDS, SDS, and RS in wheat starch samples. KC‐κ‐carrageenan; KGM‐konjac glucomannan; TA‐tannic acid; RDS‐rapidly digestible starch; SDS‐slowly digestible starch; RS‐resistant starch. Data are given as mean ± *SEM*, calculated from three replicates. Values within the column followed by different letters indicate significant difference (*p* < .05)

Compared with the control group, the digestibility rates of starch in the experimental groups were all reduced (Figure 1a). Among them, the curve of group TA was at the lowest position, which indicated that tannic acid would have a more obvious inhibition on the digestion of starch. As shown in Figure 1b, the contents of RDS in the five experimental groups were significantly lower than that in the control group, while the contents of RS were markedly increased (*p* < .05). However, the contents of SDS in each experimental group showed no striking difference, expect for group KC. In particular, due to the different ways to affect the starch digestion, group TA had the highest RS content in the experimental groups. This may be due to the interaction between tannic acid and starch (Funke & Melzig, 2005; Zhao et al., 2013). Although the difference in the content of RS between group KC and group KGM was not noteworthy, there was a remarkable difference in the contents of RDS and SDS. Specifically, the content of RDS in group KC was lower than group KGM, while the content of SDS was higher than group KGM. These may be due to the difference in the intermolecular interaction of the gel network between the two polysaccharides.

Among the three single experimental groups, the content of RDS in KGM group was lower than KC and TA group. In comparison, the content of SDS in KC group was higher than KGM and TA group, which showed that KC was more suitable for reducing postprandial blood glucose. Moreover, compared with polysaccharide groups, the combination of tannic acid and polysaccharide did not increase the variation of the three types of starch. Nevertheless, compared with the tannic acid group, the combination of tannic acid and polysaccharide markedly decreased the content of RS (*p* < .05). The same reduction in the inhibitory effect also occurred in the previous study (Sun et al., 2018). This change may be related to the polysaccharide gel hindering the direct contact of tannic acid with starch.

### 3.2 Effects of different combinations of konjac glucomannan, κ‐carrageenan, and tannic acid on the thermal properties of wheat starch

**FIGURE 2:**
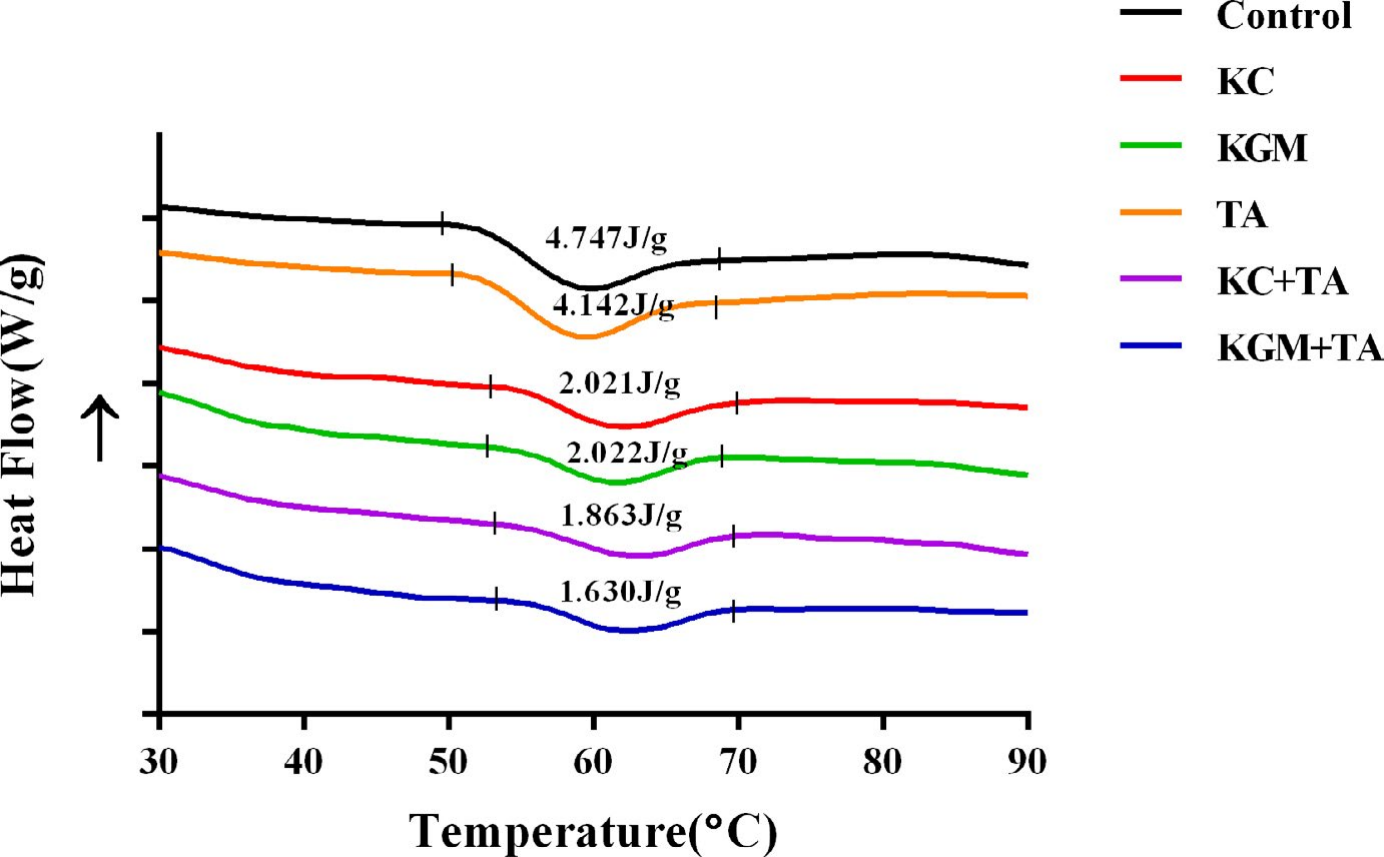
Effects of different combinations of konjac glucomannan, κ‐carrageenan, and tannic acid on thermal properties of wheat starch samples

The DSC method is commonly applied to measure the energy changes in starch, due to crystallite melting or formation of ordered structures. Table 1 showed the onset temperature (T_o_), the peak temperature (T_P_), and the gelation enthalpy (ΔH gel) obtained from the DSC thermogram (Figure 2). Compared with the control group, the four groups with dietary polysaccharides had higher gelatinization transition temperature (T_o_ and T_P_) (*p* <  .05). The increase in gelatinization transition temperature (onset, T_o_; peak, T_P_) reflected the more ordered crystallinity of starch. However, there was no significant difference between group TA and group control. Moreover, compared with group KC and group KGM, there was little influence on the transition temperatures in group KC + TA and group KGM + TA. This result demonstrated that tannic acid did not influence the gelatinization transition temperature (onset, T_o_; peak, T_P_). It was consistent with our previous study (Zeng et al., 2020).

**TABLE 1:** Thermal properties of different starch samples

Sample	*T_o_* (°C)	*T_p_* (°C)	*ΔHgel* (Jg^−1^)
Control	53.36 ± 0.19c	58.57 ± 0.13c	4.75 ± 0.12a
KC	56.68 ± 0.50a	60.75 ± 0.32b	4.14 ± 0.22b
KGM	55.55 ± 0.22b	61.41 ± 0.30ab	2.02 ± 0.07c
TA	53.59 ± 0.37c	58.44 ± 0.24c	2.02 ± 0.04c
KC+TA	56.67 ± 0.41a	61.99 ± 0.21a	1.86 ± 0.10d
KGM+TA	56.56 ± 0.32ab	62.15 ± 0.26a	1.63 ± 0.05e

Note

*n = 3, *T_o_* = onset temperature (°C); *T_p_* = peak temperature (°C);*ΔHgel* = enthalpy of gelatinization (Jg^−1^). KC‐κ‐carrageenan; KGM‐konjac glucomannan; TA‐tannic acid. All data were means of triplicates. Results are expressed as mean ± *SEM*. Means with the same letter are not significantly different (*p* > .05).

As is known to all, the gelation enthalpy (ΔH) reflects the energy intake of gelation, which is an indicator of the degree of molecular order loss in the particles during gelation (Koteswara et al., 2014). Therefore, the decrease in the gelation enthalpy (ΔH) in the experimental groups reflected the partial gelatinization of starch in the system. Compared with the single additive group, the gelation enthalpy (ΔH) of group KC + TA and group KGM + TA was further decreased, which reflected the synergistic effect of dietary polysaccharides and polyphenols in inhibiting the formation of ordered structures.

### 3.3 Effects of different combinations of konjac glucomannan, κ*‐*carrageenan, and tannic acid on the recrystallization characterization of wheat starch

**FIGURE 3:**
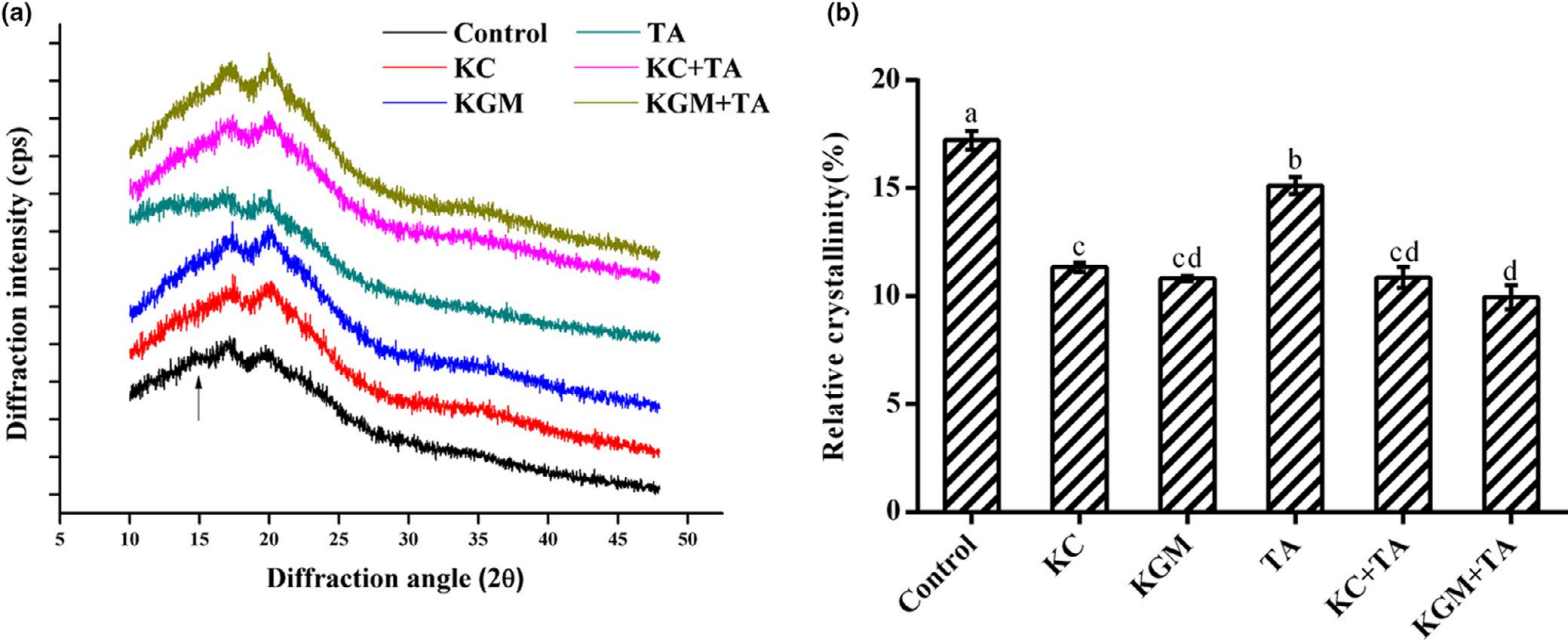
Effect of different combinations of konjac glucomannan, κ‐carrageenan, and tannic acid on X‐ray diffraction pattern of wheat starch samples. (a) XRD pattern of freeze‐dried wheat starch samples. The arrow indicates the gradually disappeared crystalline diffraction peak at 15°. (b) Relative crystallinity. Data are given as mean ± *SEM*, calculated from three replicates. Values within the column followed by different letters indicate significant difference (*p* < .05)

X‐ray diffractometry is applied to determine the crystalline structures of starch and its degree of crystallinity (Blazek & Gilbert, 2011). Many crystalline structures were formed when the starch molecules rearranged in the retrogradation process. It could be seen that the control group had diffraction peaks around 15°, 18°, and 20° at 2θ (Figure 3a), which indicated that the freeze‐dried wheat starch mainly had obvious A‐type crystals. Compared with the control group, the remaining five groups only had diffraction peaks around 18 ° and 20 °. Besides, the angle of the diffraction peaks had a slight change (Figure 3a). This means that these five groups were mixtures of A‐type crystals and V‐type crystals (Maache‐Rezzoug et al., 2011). As shown in Figure 3b, compared with control group (17.2%), the relative crystallinities of the five experimental groups were significantly reduced (*p* < .05), especially in the four groups with polysaccharides. Besides, the relative crystallinities of group KC + TA and group KGM + TA were lower than the starch with these three substances alone. This result was supported by the thermal analysis showed above. Additionally, the relative crystallinities of group KGM + TA were memorably lower than that of group KC + TA, which indicated that the inhibiting effect of KGM on starch retrogradation was better than that of KC. This discrepancy might be attributed to the interaction between the two different polysaccharide and starch.

### 3.4 Effects of different combinations of konjac glucomannan, κ*‐*carrageenan, and tannic acid on the molecular interaction characterization of wheat starch

**FIGURE 4:**
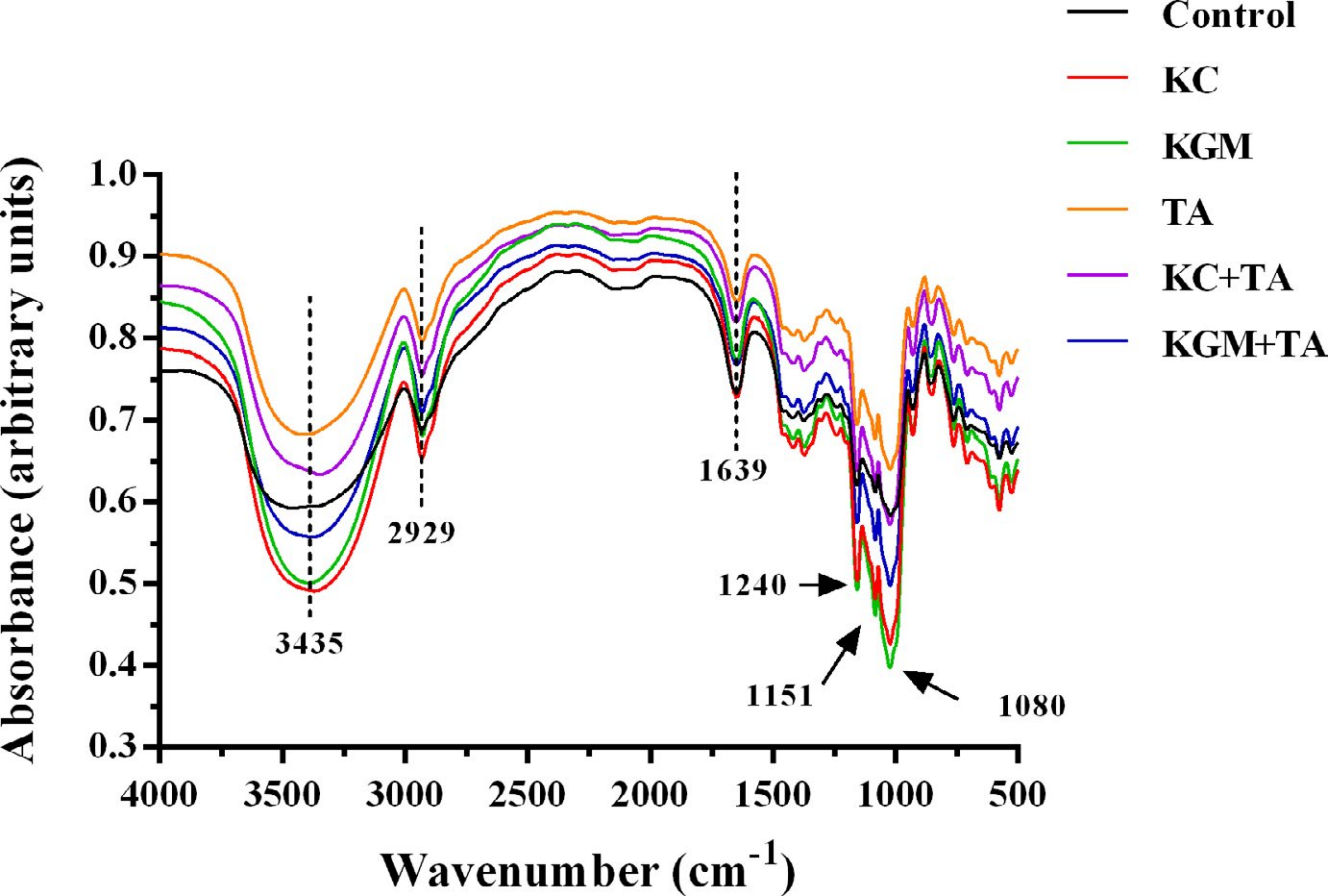
Fourier transform infrared spectroscopy of freeze‐dried wheat starch samples with different combinations of konjac glucomannan, κ‐carrageenan, and tannic acid

To have a better understanding of the molecular structure of wheat starch, FTIR is applied to analyze the functional group shifts of starch reacted with other compounds. Figure 4 represented the similar major peaks of the six samples with the variant amplitudes. These results demonstrated that the addition of polysaccharides and polyphenols to starch did not produce new functional groups (Jiang et al., 2019). However, the molecular interaction still exists among the three dietary components and wheat starch. The stretching vibration of the hydrogen bond in starch is confirmed by the wavelength of 3,435cm^−1^ (Sukhija et al., 2016). The addition of the polysaccharides increased the strength of the hydrogen bonds in starch, while tannic acid reduced the strength. When tannic acid was added to the polysaccharide group, the strength of hydrogen bonds in the system was reduced, indicating that tannic acid changed the stability of hydrogen bonds between the two polysaccharides and starch. Compared with the control group, the characteristic band at 2,929 cm^−1^ of the κ*‐*carrageenan group was strengthened, which related to the CH_2_ deformation on the carbon skeleton (Wang et al., 2019). In contrast, other experiment groups were weakened to varying degrees. The contrary effect was related to the simple linear structure of κ*‐*carrageenan, that is, it was more likely to enter the interior of starch molecules and thus interacted with the carbon skeleton (Bui et al., 2019). This was consistent with the trends of the RDS and SDS, which could be used in explaining the influence of KC on the starch digestion. Compared with the control group, the peak intensity of the experiment groups was decreased at 1639 cm^−1^, which indicated that the absorption of free water was inhibited (Dankar et al., 2018; Sukhija et al., 2016). The absorption peaks (1,240 cm^−1^, 1,151 cm^−1^, and 1,080 cm^−1^) were related to C‐O stretching, the coupling of C‐O (in the pyran ring), C‐C stretching, and C‐O‐H bending, respectively (Jiang et al., 2019). Just like the results of the wavelength at 3,435cm^−1^, the addition of the polysaccharides increased the height of peaks at these three wavelengths, while tannic acid reduced the height of peaks. When tannic acid was added to the polysaccharide group, the height of peaks in the system was reduced. All of the results indicated that tannic acid could weaken the interaction between the polysaccharides and starch molecules.

### 3.5 Effects of different combinations of konjac glucomannan, κ‐carrageenan, and tannic acid on the morphological characterization of wheat starch

**FIGURE 5:**
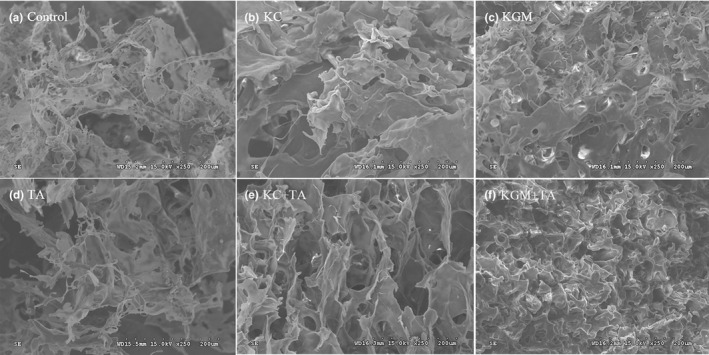
Scanning electron micrographs (*SEM*) image of freeze‐dried starch samples with different combinations of konjac glucomannan, κ‐carrageenan, and tannic acid. (a) Control (b) KC (c) KGM (d) TA (e) KC + TA (f) KGM + TA

The molecular morphology of freeze‐dried starch in the six groups was significantly different, which suggested different interactions between the additives and starch. Compared with the group control, the starch of the four groups with dietary polysaccharides formed a denser and smooth structure with smaller holes (Figure 5). When the starch was added with tannic acid, the molecular borders present more irregular crimps. As for group KC and group KGM, the molecular surface of freeze‐dried wheat starch after adding tannic acid had smaller pore diameter. Besides, the holes in wheat structure formed by konjac glucomannan were smaller than that of κ‐carrageenan. Similar results also happened between group KC + TA and group KGM + TA.

**FIGURE 6:**
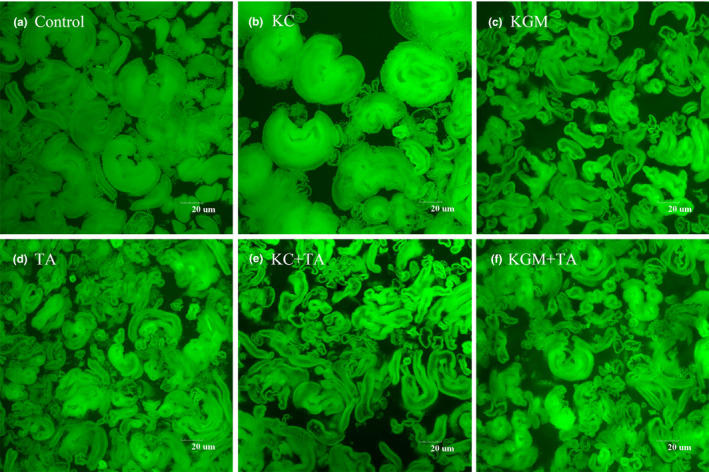
Confocal laser scanning microscopy (CLSM) image of gelatinized starch samples with different combinations of konjac glucomannan, κ‐carrageenan, and tannic acid. (a) Control (b) KC (c) KGM (d) TA (e) KC + TA (f) KGM + TA

In addition, the gelatinized starch molecules without being freeze‐dried also showed significant differences(Figure 6). Compared with control group, the starch granules in group KC were slightly enlarged. However, the starch granules in group KGM and group TA were reduced to different degrees, especially in group KGM. This indicated that these two polysaccharides had different effects on the starch structure. Compared with the two polysaccharides group, the group KC + TA and group KGM + TA significantly inhibited the expansion of starch molecules after the addition of tannic acid (Figure 6b, c, e, f). Especially, the size of the starch molecule reduced most obviously in group KC + TA.

## 4 DISCUSSIONS

Our previous study showed that tannic acid reacted with starch granules and altered their physicochemical properties (e.g., gelatinization and retrogradation) and the microstructure properties, and demonstrated tannic acid reduced the starch digestion by enzyme inhibition and the changed structures (Zeng et al., 2020). Konjac glucomannan also played an important role in the microstructure of wheat starch and its digestibility, which acted by forming a barrier around the granules (Zhang et al., 2020). Similar result was also found by κ‐carrageenan in this study. Besides, the two polysaccharides in the present study could weaken the inhibition of tannic acid on starch hydrolysis. This change may be related to the polysaccharide gel hindering the direct contact of tannic acid with starch. Moreover, FTIR results showed that tannic acid could weaken the interaction between the polysaccharides and starch molecules. These results suggested that tannic acid and polysaccharides might be competitively bound to starch. Further research found that the two polysaccharides and tannic acid had a synergistic effect on inhibiting the formation of ordered starch structure. Then, a new macromolecular network structure (tannic acid–polysaccharide**–**starch) was formed. This made the starch structure more compact and further inhibited the expansion of starch, which in turn modulated the starch digestion.

Additionally, the molecular morphology results showed that the two polysaccharides had different effects on the starch structure. This may be because that konjac glucomannan formed a stronger gel network than κ‐carrageenan, which inhibited the expansion of starch. Besides, tannic acid could increase the density and complexity of the starch structure with dietary polysaccharides, which made the starch molecules subjected to more extrusion and shear forces, and finally significantly inhibited the expansion of starch molecules (Wei et al., 2019). Moreover, the size of the starch molecule reduced most obviously in group KC + TA. This result may be due to the sulfate group in κ‐carrageenan and the phenolic hydroxyl group in tannic acid. Studies have shown that the interaction between starch and carrageenan appeared to be strongly dependent from the carrageenan charge density (Lascombes et al., 2017). It can be concluded that the addition of tannic acid increased the negative charge density in the system, which might reduce the interaction between κ‐carrageenan and starch molecules, so that more κ‐carrageenan formed a gel around starch molecules to inhibit the expansion of starch.

## 5 CONCLUSIONS

Overall, the two polysaccharides weakened the inhibition of tannic acid on starch hydrolysis. Besides, tannic acid reduced the interaction between the polysaccharides and starch molecules. These results suggested that tannic acid and polysaccharides starch might be competitively bound to starch. Further research found that the two polysaccharides and tannic acid had a synergistic effect on the formation of ordered starch microstructure, which made the starch structure more compact and inhibited the expansion of starch. Additionally, tannic acid strengthened the microstructure of the starch with dietary polysaccharides. The understanding of the starch microstructure caused by the two polysaccharides, and tannic acid could be useful for explaining the starch digestion.

## CONFLICT OF INTEREST

There is no potential conflict between authors and others that bias our work.

## AUTHOR CONTRIBUTIONS

**juncheng he:** Writing‐original draft (lead). **lirong zeng:** Writing‐original draft (supporting). **junan gong:** Investigation (equal). **yalun he:** Methodology (equal). **xiong liu:** Data curation (equal). **ling zhang:** Data curation (equal); Supervision (equal). **na xu:** Writing‐review & editing (supporting). **qiong wang:** Writing‐review & editing (lead).

## ETHICAL APPROVAL

This study does not involve any human or animal testing.

## Data Availability

The data that support the findings of this study are available from the corresponding author upon reasonable request.
